# Simulation degradation datasets for health prognosis of a control moment gyroscope’s flywheel system

**DOI:** 10.1016/j.dib.2026.112877

**Published:** 2026-05-20

**Authors:** Diyin Tang, Siyuan Liang, Danyang Han, Bin Chen, Jinsong Yu

**Affiliations:** a School of Automation Science and Electrical Engineering, Beihang University, Beijing 100191, China; b Hangzhou International Innovation Institute, Beihang University, Hangzhou 311115, China; c Beijing Control Engineering Institute, Beijing 100094, China

**Keywords:** Simulation data, Prognostics and health management, Space environment, Brushless dc motor

## Abstract

A Control moment gyroscope (CMG) is an attitude control actuator widely used in spacecraft. As a consequence of the continuous high-speed rotation to output stable angular momentum, CMGs are at a higher risk of degradation failure, which could lead to a loss of spacecraft control. However, implementing health prognosis for CMGs is challenging due to the high cost associated with conducting comprehensive lifetime tests. Moreover, environment differences between space and ground make data generated with simulation in the space environment more valuable than data from ground experiments for health prognosis. Thus, we develop a simulation model for the flywheel system of a CMG to generate degradation data, aiming to advance deep learning-based health prognosis in aerospace applications. The datasets focus on the brushless DC motor of the flywheel system, encompassing the bus current, bus voltage, rotor speed time window from CMGs under three different operational conditions. A data-approximate model is built and two parameters are set to change over time to simulate the mechanical-loss-related and electromechanical-conversion-related degradation. When compared to a dataset from a real CMG, the simulation dataset demonstrates similar dynamic characteristics and data patterns. The dataset can support the development and validation of prognostics methods such as remaining useful life(RUL) prediction, degradation trend estimation, and cross-condition transfer learning for CMG flywheel systems.

Specifications Table

**Table:** 

Subject	Engineering & Materials science
Specific subject area	The dataset contains sensor data of degrading CMG flywheel subsystems generated from a data-approximate simulation model.
Type of data	Raw
Data collection	The data were generated using a Simulink model of a CMG flywheel subsystem. Three observable variables (current, voltage, and rotational speed) and four parameters were recorded. Two degradation-related parameters, $B_{m}$ and $K_{t}$, were evolved over time to simulate degradation. The data are stored as .mat files.
Data source location	Institution: Beihang University City/Town/Region: Beijing Country: China
Data accessibility	https://data.mendeley.com/datasets/52d635b8yp/2 Instructions for accessing data: Data is .mat file
Related research article	None

## 1 Value of the Data

• This dataset is collected from numerical simulations of a MATLAB/Simulink model. The model is developed based on the characteristics of a CMG’s flywheel system and exhibits similar response properties. Consequently, this model can help analyze the dynamic behavior of a CMG's flywheel system.
• The dataset contains current, voltage, and rotational speed sensor data. The placement of these sensors is consistent with that in an operational CMG, and the degradation characteristics align with those observed in ground-based accelerated degradation tests. Based on this, the dataset can support the development and validation of health prognosis methods, such as RUL prediction and degradation trend estimation.
• The dataset comprises data from three different operational conditions. The rotational speed command sequence varies for each condition, leading to differences in data distribution. The data specifications are detailed in Table 1. Using data from one condition as the source domain and others as the target domain enables the validation of transfer-learning methods.

**Table 1:** Size description of dataset. Each sample (folder name 001, etc.) in an operational condition consists of multiple cycles in mat format. The sample rate is set to 5µs.

Condition	Size	Number of Cycles in Each Sample
OC1	25	[189,193,158,159,190,172,165,176,172,189,182,169,184,148,163,171,180,182,164,190,182,166,166,190,189]
OC2	5	[147,141,139,162,140]
OC3	5	[212,205,235,205,228]
• Publicly available degradation datasets for CMGs are still very limited. This dataset therefore provides a useful resource for prognostics and health management research on aerospace electromechanical systems.

## 2 Background

CMGs are the essential key actuator for three-axis stabilized spacecraft in near-Earth orbit because of their well-known torque amplification property [1]. It provides high-speed attitude control with high precision and stability [2] and more capacity of the spacecraft using Zero Propellant Maneuver (ZPM) [3]. Thus, the performance degradation of CMGs always influences the spacecraft a lot, even leads to an overall damage. For example, there are only three CMGs working in the international space station (ISS) as two failures of CMG occurred, which postponed the space exploration plan for one year [4].

Therefore, it is necessary to utilize prognostics and health management (PHM) technology in the early stage of the CMG’s degradation. Considering the system complexity, PHM method for CMGs mainly focuses on the data-driven method, which requires numerous monitoring data. However, real operational data are extremely scarce due to the prohibitive cost of conducting sufficient reliability tests. And existing models of CMG mainly concentrate on the control design without enough detail to support fault injection.

Thus, our data is extracted from a Simulink model of CMGs with manual degradation injection, aiming at health prognosis of CMG. The dataset can be used to support the development and validation of health prognosis models, including RUL prediction, degradation trend estimation, and cross-condition transfer learning. In particular, OC1 can be used as a source domain and OC2/OC3 as target domains for cross-domain prognostics studies.

## 3 Data Description

This dataset comprises three folders (OC1, OC2, and OC3), corresponding to three distinct operational conditions. As summarized in Table 2, the dataset contains 25 samples for OC1 and 5 samples each for OC2 and OC3.

**Table 2:** The variables in each mat file.

Condition	Sample Size	Rotational Speed(rpm)		Load Torque(N·m)	
OC1	25	3000		1.0	
Sample No.	Cycles	B_m_(10–3 Nm/rad/s)	K_t_(V/rad/s)	Sample interval(µs)	Time Per cycle(s)
001	189	0.93115→3.016	1.4147→1.1094	5	3
002	193	0.99054→3.114	1.4119→1.0465	5	3
003	158	0.88812→2.968	1.3946→1.1316	5	3
004	159	1.08737→3.071	1.3784→1.1071	5	3
005	190	1.03584→3.063	1.3942→1.0800	5	3
006	172	1.00637→3.037	1.3977→1.0781	5	3
007	165	1.02368→3.001	1.3904→1.1072	5	3
008	176	0.87467→3.081	1.4042→1.0667	5	3
009	172	1.04268→3.079	1.3974→1.1064	5	3
010	189	0.99198→2.998	1.3816→1.0495	5	3
011	182	1.09805→2.967	1.4061→1.1084	5	3
012	169	0.93577→3.015	1.3956→1.1097	5	3
013	184	1.01020→2.960	1.4044→1.0100	5	3
014	148	1.02560→2.995	1.4153→1.1743	5	3
015	163	1.03082→3.000	1.4093→1.1370	5	3
016	171	1.04515→2.984	1.3772→1.0638	5	3
017	180	1.04909→3.065	1.4110→1.1065	5	3
018	182	1.00897→3.049	1.3892→1.0864	5	3
019	164	1.05250→3.011	1.3996→1.0953	5	3
020	190	0.96252→3.030	1.4136→1.1232	5	3
021	182	0.96140→3.012	1.4119→1.0735	5	3
022	166	0.92257→2.967	1.4135→1.1366	5	3
023	166	0.88090→3.019	1.3795→1.0960	5	3
024	190	0.86178→3.082	1.4057→1.0825	5	3
025	189	0.92800→3.021	1.4141→1.0703	5	3

Condition	Sample Size	Rotational Speed(rpm)		Load Torque(N·m)	
OC2	5	3150		1.0	
Sample No.	Cycles	B_m_(10–3 Nm/rad/s)	K_t_(V/rad/s)	Sample interval(µs)	Time Per cycle(s)

001	147	1.06933→2.999	1.4053→1.1055	5	3
002	141	1.01491→3.023	1.3752→1.0910	5	3
003	139	0.95347→3.004	1.4010→1.0957	5	3
004	162	1.02909→3.008	1.4077→1.0561	5	3
005	140	1.01509→2.979	1.3983→1.1031	5	3

Condition	Sample Size	Rotational Speed(rpm)		Load Torque(N·m)	
OC3	5	3150		0.5	
Sample No.	Cycles	B_m_(10–3 Nm/rad/s)	K_t_(V/rad/s)	Sample interval(µs)	Time Per cycle(s)

001	212	0.99048→3.081	1.3803→1.0223	5	3
002	205	1.03401→3.077	1.4069→1.1316	5	3
003	235	1.02182→3.052	1.4083→1.0483	5	3
004	205	1.00678→3.001	1.4297→1.1742	5	3
005	228	1.11563→3.038	1.3888→1.0987	5	3

Each sample is stored in an independent folder, capturing the complete degradation process of the CMG flywheel subsystem from an initial healthy state to defined end-of-life criterion. These sample folders are sequentially named (from 001 to 025). Within each folder, data from multiple cycles are stored in “.mat” files, named according to the pattern “{Cycle Number}.mat”. Each cycle simulates 3 s of operation with a 5 µs time step, resulting in 600,002 data points per variable sequence.

Each data file contains three time-series data (voltage u, current i, and rotational speed w) and four parameters (the degradation parameters B and Kt, a fixed torque T, and the load torque TL). A detailed description is provided in Table 3. It is noteworthy that OC2 operates at a different rotational speed, while OC3 incorporates variations in both rotational speed and load torque. This design intentionally introduces composite domain shifts between OC1 (the source domain) and OC2/OC3 (the target domains). Consequently, this dataset is particularly suited for validating RUL prediction algorithms and is highly effective for assessing the comprehensive performance of transfer learning algorithms in cross-condition prognostics(OC1→OC2 or OC1→OC3).

**Table 3:** The variables in each mat file.

Name	Size	Meaning	Unit
B	1 × 1,double	Viscous damping coefficient, increasing over time	Nm/rad/s
i	60,002×1,double	Current of the rotor	A
Kt	1 × 1,double	Torque coefficient, decreasing over time	V/rad/s
T	1 × 1,double	Torque of the flywheel system, fix at 3.2	N·m
TL	1 × 1,double	Load Torque of the flywheel system	N·m
u	60,002×1,double	Voltage of the rotor	V
w	60,002×1,double	rotational speed of the rotor	Rad/s

## 4 Experimental Design, Materials and Methods

A typical CMG mainly consists of a gimbal system and a flywheel system [5]. The gimbal system, comprises a gimbal motor with a gearhead, a bracket and a slip ring, rotates the whole flywheel assembly to output angular torque and spin the direction of the angular momentum. Meanwhile, a flywheel and its motor, forming flywheel system, create the angular momentum. By controlling the gimbal system, angular momentum is exchanged between the spacecraft and the rapidly rotating flywheel. This enables the spacecraft to adjust its attitude rapidly and accurately. However, regardless of whether attitude adjustment is required, the flywheel subsystem usually operates continuously at high speed [6]. Consequently, the main faults in CMGs derive from mechanical wear of gears or failures of the motor within the flywheel system. Thus, this work focuses on the flywheel subsystem to generate a degradation-oriented dataset.

Given their outstanding dynamic characteristics, precision, and ability to adapt to severe environments, Brushless DC (BLDC) motors are typically chosen to drive the rotor in the flywheel system. In the spacecraft, the power supply system provides a DC source. Then, after an inverter, the motor is driven by the three-phase voltage. Meanwhile, the Hall sensor samples the position and sends it into the controller. The controller generates the gate signal using the set rotational speed and decoded data from Hall sensors. As the gate signal determines the frequency of the inverter, the rotational speed can be controlled in this closed-loop way.

**Fig. 1:**
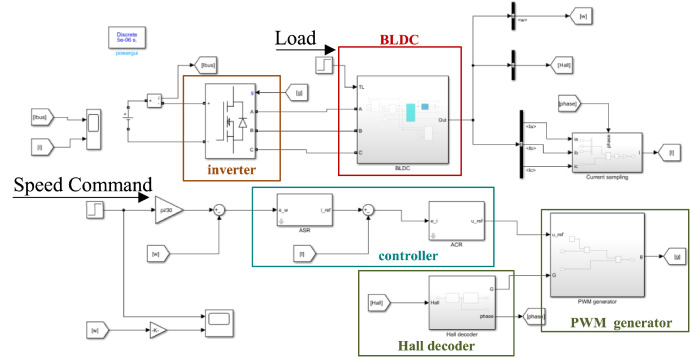
The whole Simulink model of the flywheel system of the CMG.

According to the mentioned process, the Simulink model of the flywheel system can be divided into an inverter, a BLDC, a controller, a Hall encoder and a PWM generator, as shown in Fig 1. The marks and units of key parameters are shown in Table 4.

**Table 4:** Key parameters of the flywheel system.

Type	Symbol in Equation	Unit	Description
Working condition	$n_{0}$	rad/s	Speed reference of rotor
	$T_{L}$	N·m	Load of the flywheel system
Observable variable	$I_{S}$	A	Bus current of BLDC
	$U_{S}$	V	Bus voltage of BLDC
	$n_{S}$	rad/s	Rotor speed
Unobservable variable	$U_{a},U_{b},U_{c}$	V	Terminal voltage of three phases
	$I_{a},I_{b},I_{c}$	A	Line current of three phases
	$e_{a},e_{b},e_{c}$	V	Back electromotive force (EMF) of three phases
Implicit state variable	$K_{t}$	V/rad/s	Electric potential constant
	$B_{m}$	N·m/rad/s	Viscous damping coefficient

### 4.1 Model of the inverter

In the inverter, six switches work in concert under the control of the gate signal g to output the voltage A, B, C. Simulink has offered a standardized module named universal bridge, with which we can easily realize the inverter by setting the Number of bridge arms to 3.

### 4.2 Model of the BLDC

**Fig. 2:**
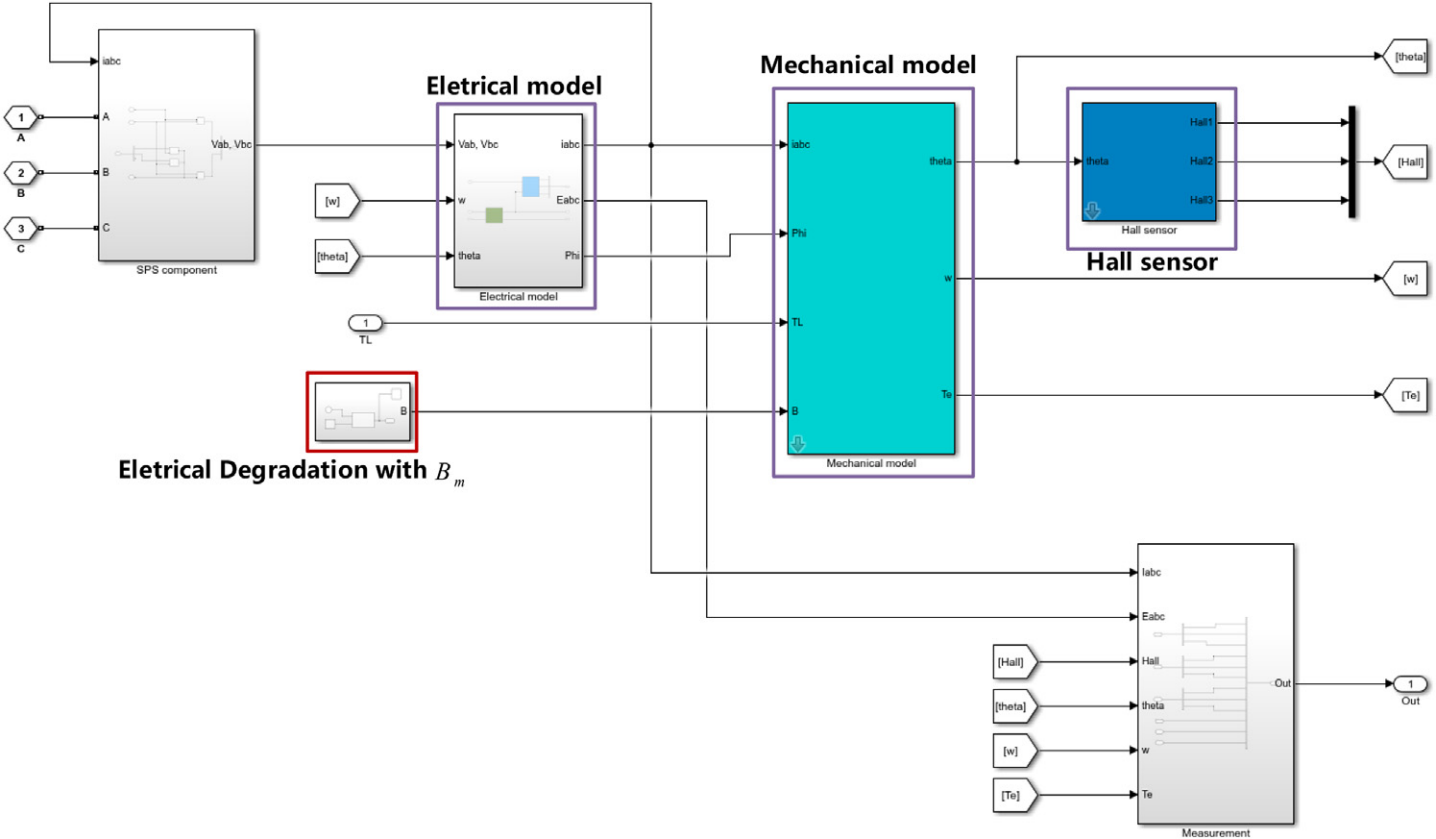
Model of the block *BLDC.*

BLDC takes voltage A,B,C as input and the load torque $T_{L}$ is reserved to adjust in different work conditions. The model of BLDC outputs the rotational speed ω, code from Hall sensors and load current I. Although there are module Permanent Magnet Synchronous Machine (PMSM) in Simulink, we can’t change some built-in parameters to simulate the degradation of existing black box module. Thus, we rebuild a BLDC through an electric model, a mechanical model, Hall sensors, shown in Fig 2.

### 4.3 Electrical model

**Fig. 3:**
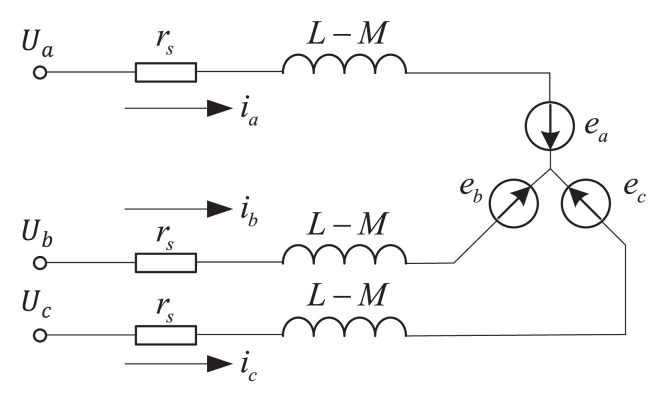
Equivalent circuit for the windings of BLDC.

**Fig. 4:**
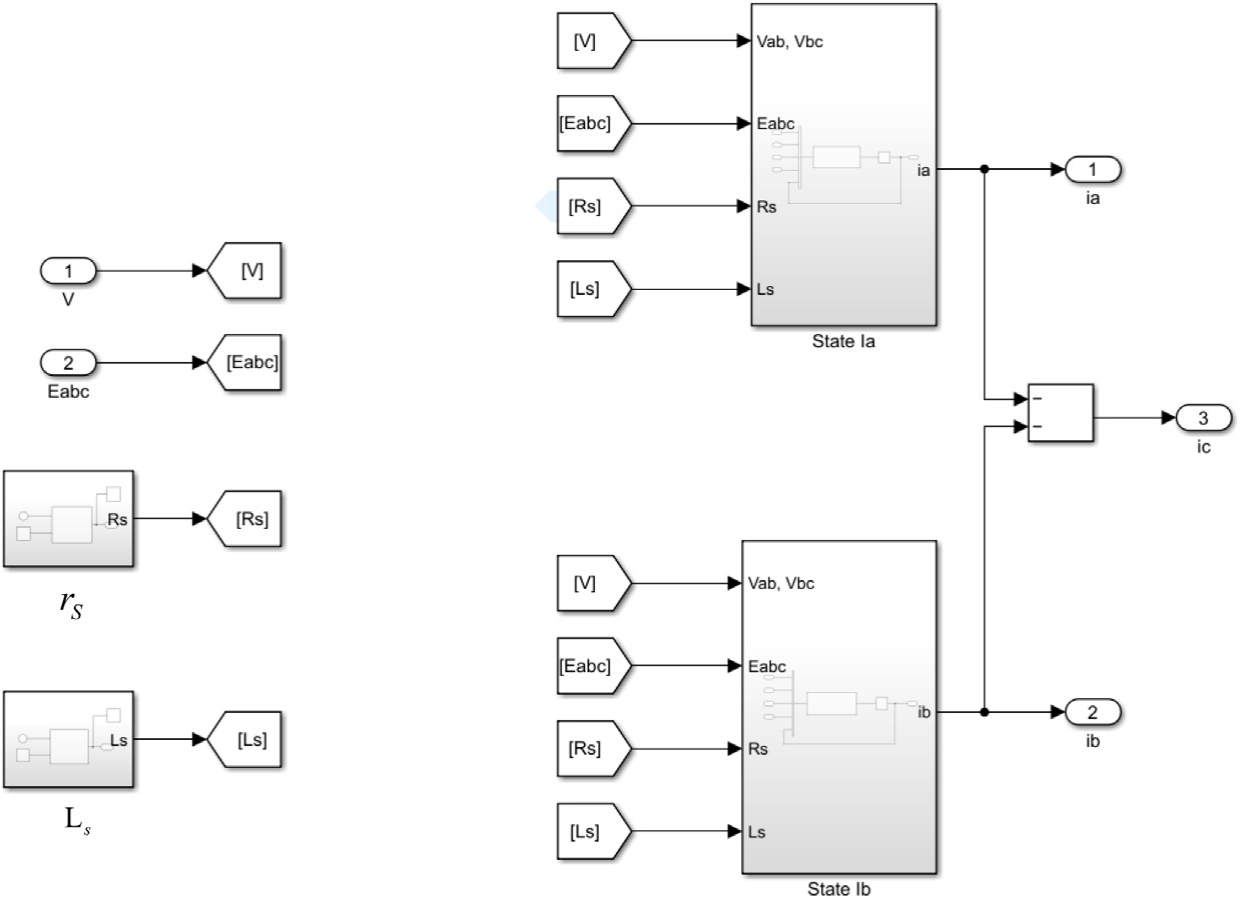
Model of the block *current calculation.*

The equivalent circuit of the stator of BLDC is shown in Fig 3, where $e_{a},e_{b},e_{c}$denotes back EMF, LandMdenote self and mutual inductance by assuming $L_{a}=L_{b}=L_{c}=L$ and $L_{ab}=L_{ba}=L_{ca}=L_{ac}=L_{bc}=L_{cb}=M$. Applying Kirchhoff’s law, the main differential equation of the electric model is Eq. (1).

(1) $$\left\{ \begin{array}{c} \frac{di_{a}}{dt}=\frac{2v_{ab}+v_{bc}-3r_{S}i_{a}-2e_{a}+e_{b}+e_{c}}{3L_{S}}\\ \frac{di_{b}}{dt}=\frac{-v_{ab}+v_{bc}-3r_{S}i_{b}+e_{a}-2e_{b}+e_{c}}{3L_{S}}\\ \frac{di_{c}}{dt}=-\frac{di_{a}}{dt}-\frac{di_{b}}{dt} \end{array}\right.$$

where $v_{ab}=v_{a}-v_{b},v_{bc}=v_{b}-v_{c}$ and $L_{S}=L-M$. Accordingly, we establish the block *current calculation* in Simulink as Fig 4, where *State Ia* and *State Ib* solve the first two differential equations.

Ideally, the back EMF is trapezoidal wave as Eq. (2).

(2) $$\left\{ \begin{array}{c} e_{a}=K_{t}f\left(\theta \right)\omega_{r}\\ e_{b}=K_{t}f\left(\theta -\frac{2\pi }{3}\right)\omega_{r}\\ e_{c}=K_{t}f\left(\theta +\frac{2\pi }{3}\right)\omega_{r} \end{array}\right.$$

where $K_{e}$ denotes the electric potential constant, θ denotes the electrical angle, $\omega_{r}$ denotes the electrical angular velocity and f(θ) can be descripted with piecewise linear function as Eq. (3):

(3) $$f\left(\theta \right)=\left\{ \begin{array}{c} \frac{6\theta }{\pi },0\leq \theta \leq \frac{\pi }{6}\\ 1,\frac{\pi }{6}\leq \theta \leq \frac{5\pi }{6}\\ 6-\frac{6\theta }{\pi },\frac{5\pi }{6}\leq \theta \leq \frac{7\pi }{6}\\ \begin{array}{c} -1,\frac{7\pi }{6}\leq \theta \leq \frac{11\pi }{6}\\ \frac{6\theta }{\pi }-12,\frac{11\pi }{6}\leq \theta \leq 2\pi \end{array} \end{array}\right.$$

And the relation between the mechanical angle *θ_m_* and the electrical angle *θ* is shown in Eq. (4):

(4) $$\left\{ \begin{array}{c} \omega_{r}=p\Omega_{m}\\ \theta =p\theta_{m}\\ \theta_{m}=\int \Omega_{m}dt \end{array}\right.$$

where p denotes the pole number, $\Omega_{m}$ denotes the mechanical angle velocity.

**Fig. 5:**
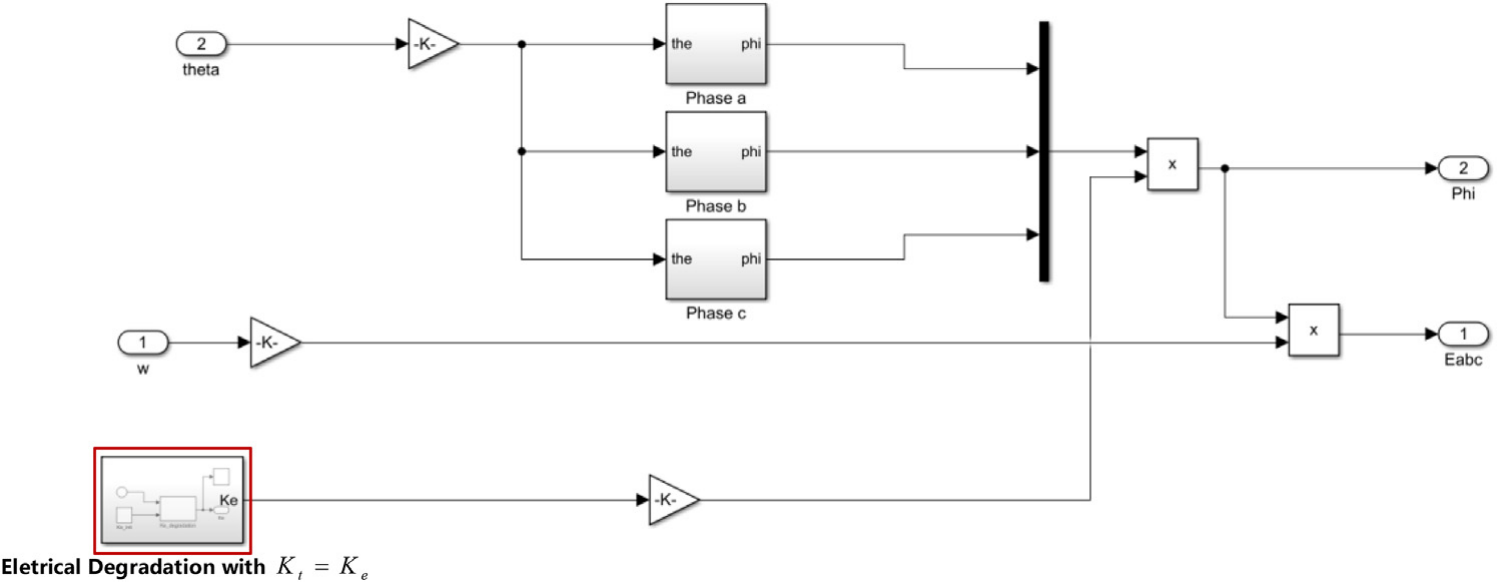
Model of the block *back EMF.*

Therefore, the model of the back EMF in Simulink is built as Fig 5 named *back EMF calculation* block, where *Phase a, Phase b, Phase c* realize f(θ),f(θ−2π/3),f(θ+2π/3) and the mechanical parameters *theta, w* are multiplied by a constant to transfer into the electrical one.

**Fig. 6:**
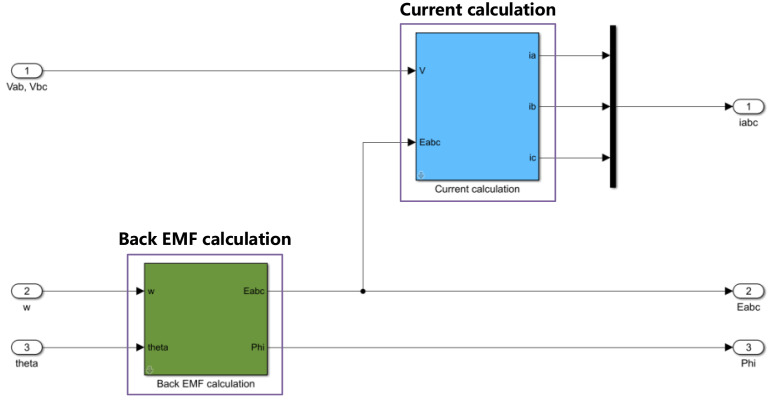
Model of the block *electrical model.*

With block *current calculation* and *back EMF calculation*, the block electrical model can output the armature current, electrical angle and back EMF with voltage, mechanical rotational speed and angle as input, as it’s shown in Fig 6.

### 4.4 Mechanical model

The kinetic equation of BLDC is Eq. (5).

(5) $$T_{em}-T_{L}=J\frac{d\Omega_{m}}{dt}+B_{m}\Omega_{m}$$

where $T_{em}$ denotes the electromagnetic torque, $T_{L}$ denotes the load torque, J denotes the moment of inertia of the BLDC, $B_{m}$ denotes viscous damping coefficient.

As $T_{em}$is generated with the interaction between the stator current and magnetic field, it can be expressed as Eq. (6).

(6) $$T_{em}=\frac{e_{a}i_{a}+{e_{b}}_{i}b+e_{c}i_{c}}{\Omega_{m}}$$

**Fig. 7:**
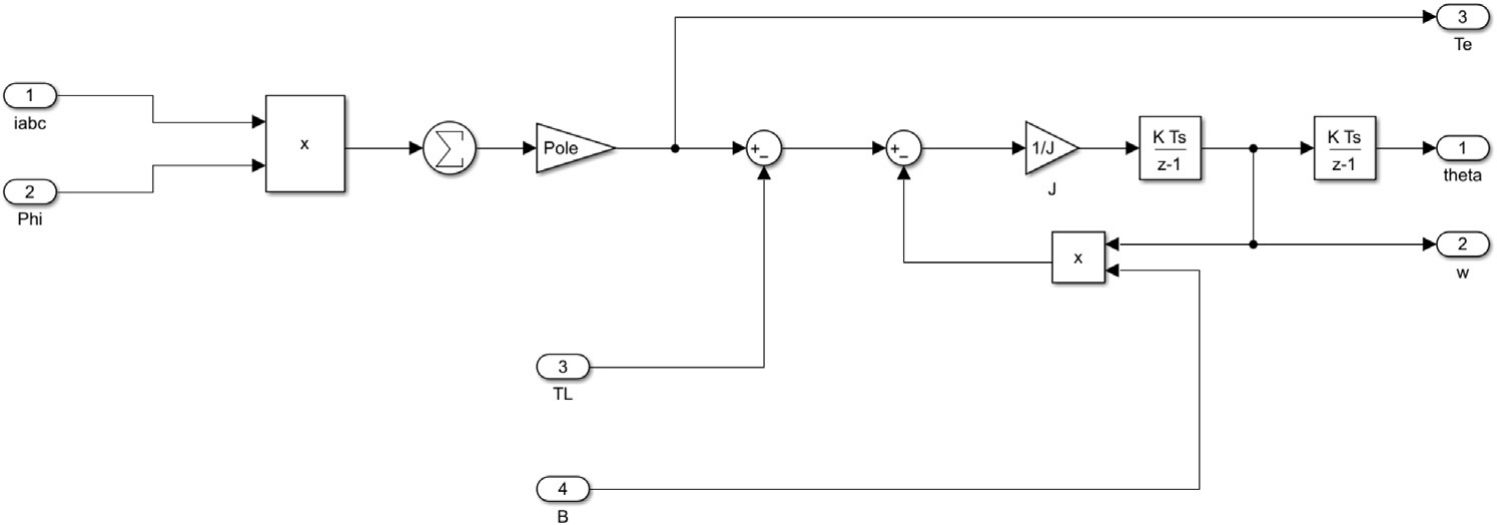
Model of the block *mechanical model.*

Accordingly, the mechanical model in Simulink is built as Fig 7.

### 4.5 Hall sensor

**Fig. 8:**
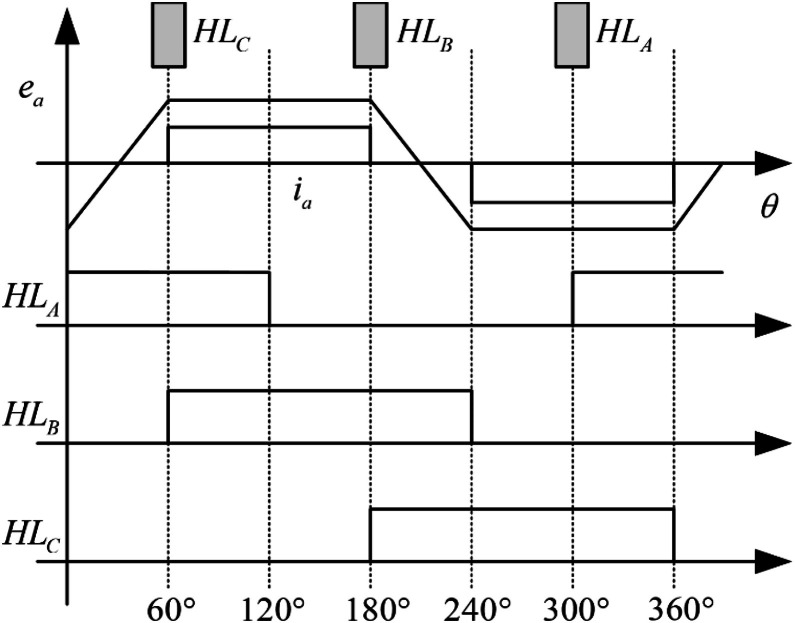
The relation between Hall signal and installation location, $e_{a}$, $i_{a}.$

In general, three Hall sensors are set every 120° in the stator [7] of flywheel in CMG to detect the position of the stator. The installation location, $e_{a},i_{a}$ and the output of the Hall sensors are shown in Fig 8.

**Fig. 9:**
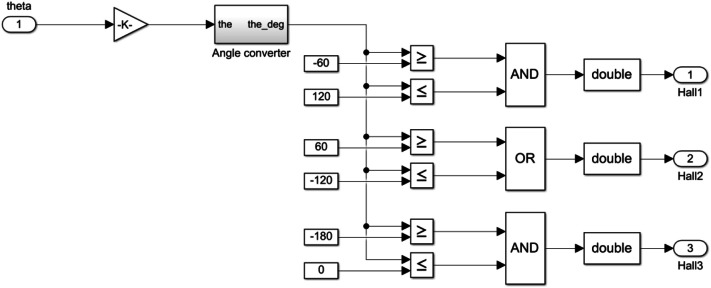
Model of the block *Hall sensor.*

Thus, the model of the Hall sensors in Simulink is built as Fig 9.

### 4.6 Model of the controller

**Fig. 10:**
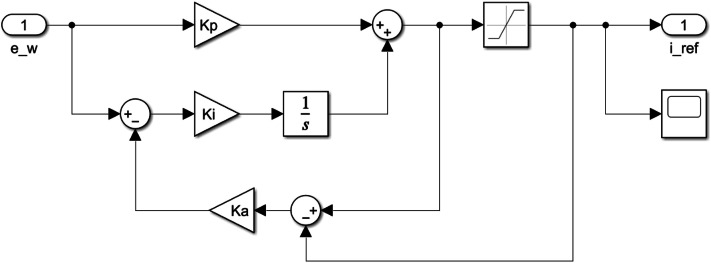
Model of the block *ASR*. As for the *ACR*, change input port *e_w* into *e_i* and output port *i_ref* into *u_ref*.

Given the target rotational speed, a speed loop and a current loop are designed to improve the dynamic characteristics. The DC Bus voltage is constant in spacecraft, as a consequence, there is saturation before the controller output the reference voltage. Different input speed variations shapes are proposed as well as extreme scenarios where the linear behavior of the PID regulator is challenged by applying fast and high magnitude speed variations so that the PID controller is not able to correctly follow the reference [8]. Thus, the controller employs a control law named back calculation for anti-windup in both speed loop and current loop. Fig 10 shows the Simulink model of automatic speed regulator (ASR) and automatic current regulator (ACR). Referring to [9], the parameters of the ACR can be decided by Eq. (7).

(7) $$\left\{ \begin{array}{c} K_{pc}=L_{S}•\omega_{cc}\\ K_{ic}=r_{S}•\omega_{cc}\\ K_{ac}=1/K_{pc} \end{array}\right.$$

where $\omega_{cc}$ denotes the cut-off frequency of ACR. In general, the switching frequency of the inverter ranges, denoted by $f_{sw}$, from 1/10 to 1/20, lower than the sample rate 1/25, thus, $\omega_{cc}$ can be chosen as Eq. (8).

(8) $$\omega_{cc}=\sqrt{\frac{1}{200}}•2\pi •f_{sw}$$

Similarly, the parameters of the ASR are decided by Eq. (9).

(9) $$\left\{ \begin{array}{c} K_{ps}=\frac{J\omega_{cs}}{K_{t}}\\ K_{is}=K_{ps}•\frac{\omega_{cs}}{5}\\ K_{as}=\frac{1}{K_{ps}} \end{array}\right.$$

where the cut-off frequency of ASR $\omega_{cs}=0.15\omega_{cc}$, as is should be lower than 1/5 of the $\omega_{cc}$.

### 4.7 Model of hall decoder

**Fig. 11:**
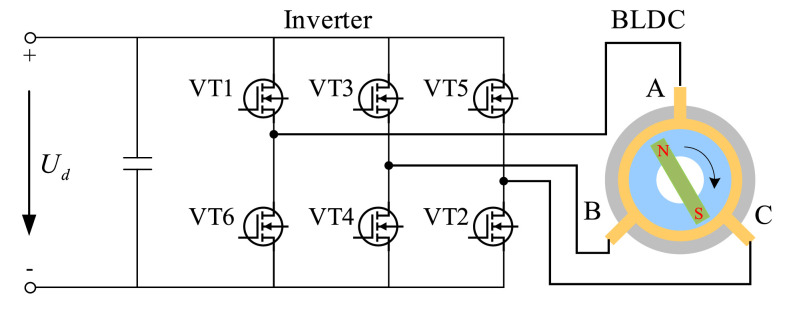
Circuit of the inverter and BLDC.

**Fig. 12:**
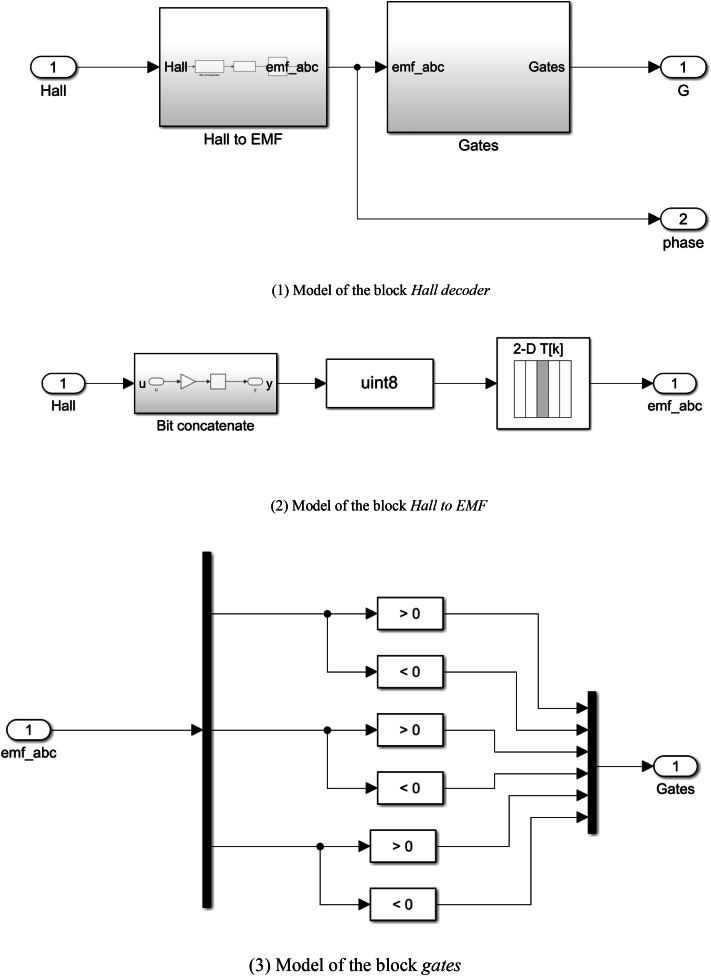
Model of the block *Hall decoder.*

The decoder for Hall sensors transfers the Hall signal into the gate signal to control the switching sequence of the switches. According to the Table 5 and Fig 11, the model of the decoder for Hall sensors is built as Fig 12.

**Table 5:** Relation between switches and pole position signal.

Conductive switch	$Hall_{A}$	$Hall_{B}$	$Hall_{C}$
$VT_{1},\;VT_{2}\left(i_{a}^{+},\;i_{c}^{-}\right)$	1	0	0
$VT_{2},\;VT_{3}\left(i_{b}^{+},\;i_{c}^{-}\right)$	1	1	0
$VT_{3},\;VT_{4}\left(i_{a}^{-},\;i_{b}^{+}\right)$	0	1	0
$VT_{4},\;VT_{5}\left(i_{a}^{-},\;i_{c}^{+}\right)$	0	1	1
$VT_{5},\;VT_{6}\left(i_{b}^{-},\;i_{c}^{+}\right)$	0	0	1
$VT_{6},\;VT_{1}\left(i_{a}^{+},\;i_{b}^{-}\right)$	1	0	1

### 4.8 Model of the PWM generator

**Fig. 13:**
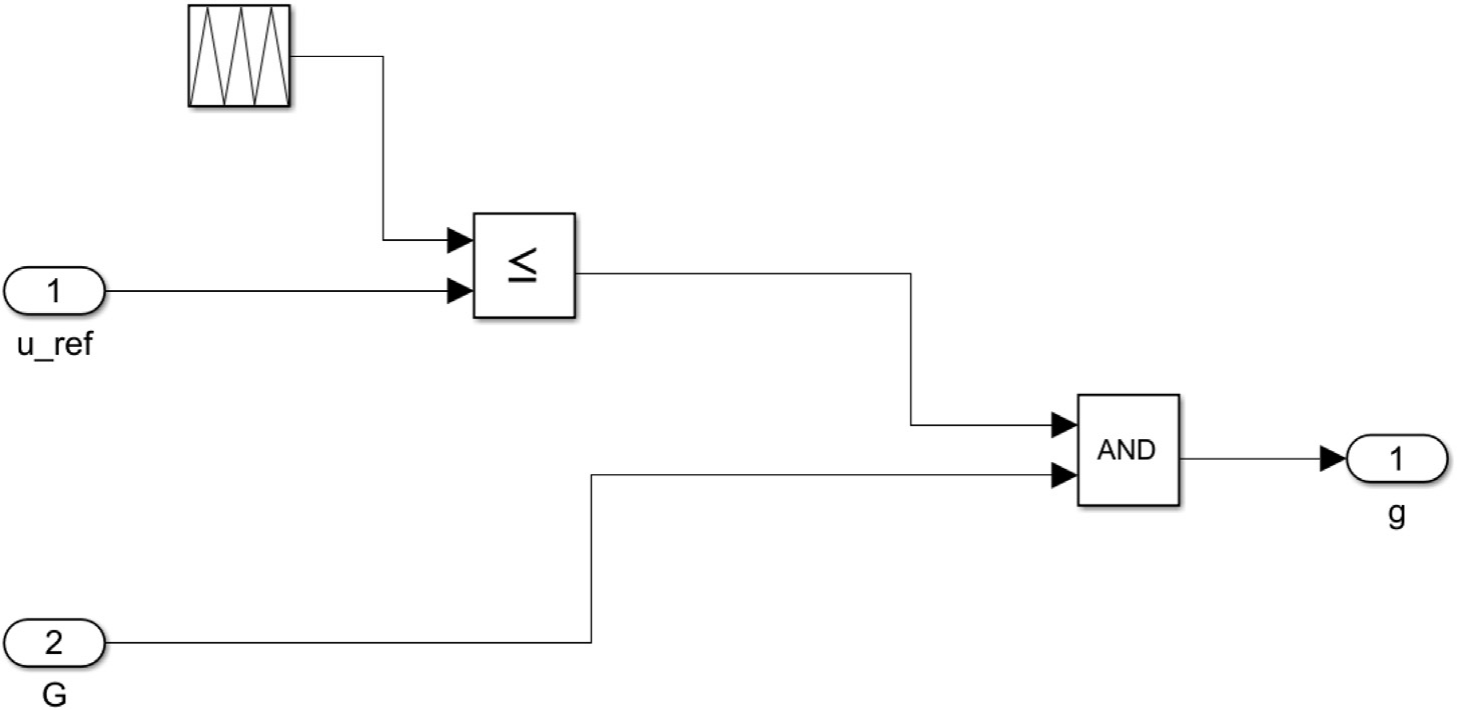
Model of the block *PWM generator.*

PWM generator compares a triangular wave and a referenced voltage to output PWM signal with different duty ratio. And the gate signal from the Hall decoder ensures commutating at the right position. The Simulink model is shown in Fig 13.

### 4.9 Validation for the model

**Fig. 14:**
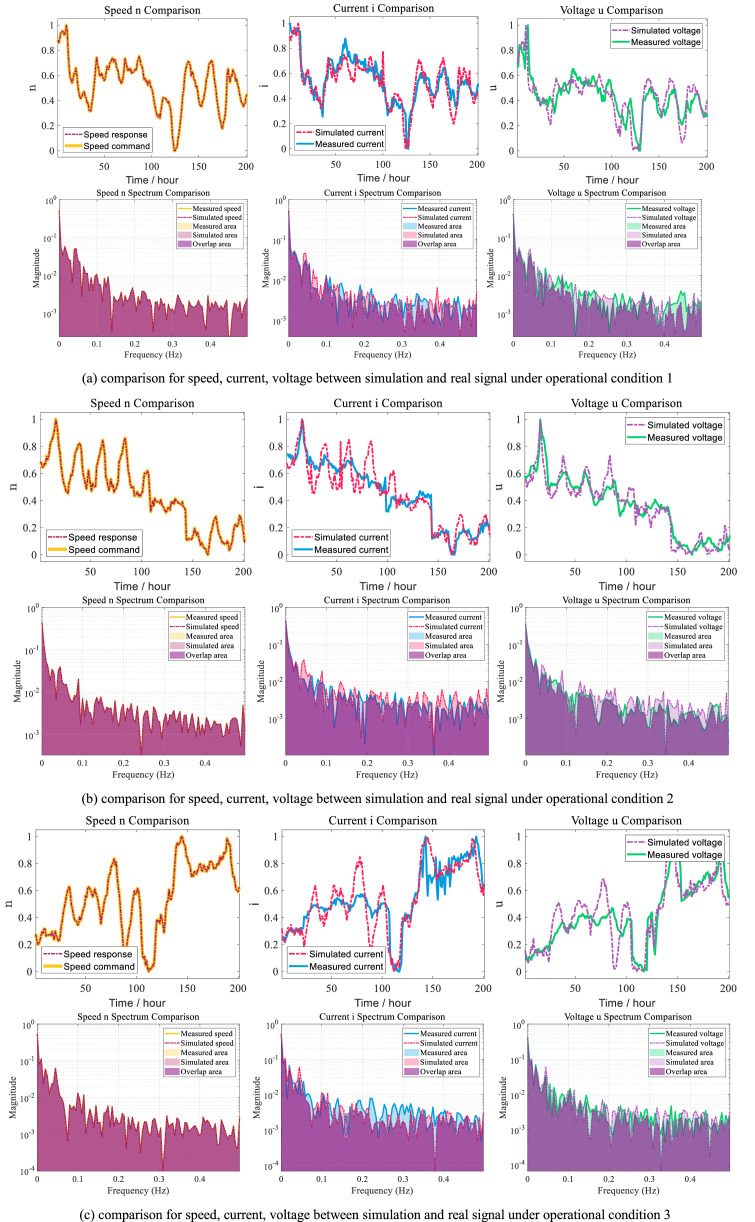
Some examples of the simulation data and the real data of CMG.

To validate the proposed simulation model, we compare its outputs with data from a real CMG ground test. The experimental dataset encompasses three distinct operational conditions: gimbal oscillation at 60°/s, constant-speed rotation at 1°/s, and oscillation at 20°/s. The measured speed profiles from these conditions are used as the speed command inputs for our simulation. The resulting current and voltage outputs from the model are then compared with their real-world counterparts, as visually summarized in Fig 14 and quantitatively assessed in Table 6. Please note that the actual test data is not publicly available due to commercial confidentiality; therefore, the comparative plots present normalized values. According to the spectrum comparison, the simulated data show lower agreement with the real data in some high-frequency components, which may be attributed to the absence of sensor noise modeling and the neglect of certain external environmental factors. However, in the low-frequency signals that are most relevant to lifetime degradation, our model shows a high degree of consistency with the actual product.

**Table 6:** Comparison for the experimental data and simulation data. Mean Absolute Error (MAE), Root Mean Square Error (RMSE) and Spectrum similarity(cosine similarity of spectrum) are used to evaluate the similarity.

Error	Operational condition 1			Operational condition 2			Operational condition 3		
	MAE	RMSE	Spectrum similarity	MAE	RMSE	Spectrum similarity	MAE	RMSE	Spectrum similarity
Current/A	0.0677	0.0863	0.9973	0.0741	0.0968	0.9956	0.0839	0.1159	0.9956
Speed/rpm	$4.8\times 10^{-5}$	$5.9\times 10^{-5}$	1.0000	$4.5\times 10^{-5}$	$5.4\times 10^{-5}$	1.0000	$4.8\times 10^{-5}$	$5.7\times 10^{-5}$	1.0000
Voltage/V	0.0797	0.1003	0.9968	0.0616	0.0774	0.9960	0.0923	0.1249	0.9946

As shown in Fig 14, the simulated current and voltage waveforms show a close alignment with the experimental data. Some discernible discrepancies are primarily caused by the following factors:

1. **Input Approximation:** We intentionally introduced minor disturbances into the speed command instead of using an ideal signal. As shown in Fig 14, despite significant speed oscillations (left panels of Fig 14a-c) and the consequent delayed and somewhat irregular response in the simulation, the overall dynamic performance remains robust and aligns well with real-world behavior.
2. **Normalization:** While the normalization process amplifies the visual prominence of errors by reducing the vertical scale, the quantitative error analysis presented in Table 6 confirms that the discrepancies between the simulated and actual responses are minimal.

In summary, using the equivalent input, the proposed simulation model demonstrates satisfactory similarity with the real CMG’s flywheel system. These results indicate that the model is sufficiently consistent with real data in the degradation-relevant trend components, thereby providing a reasonable foundation for degradation-oriented dataset generation.

### 4.10 Degradation modeling

The degradation of the flywheel subsystem in a CMG can be broadly categorized into two aspects, mechanical-loss-related and electromechanical-conversion-related [10]. In this work, we use two system-level equivalent degradation parameters, $B_{m}$ and $K_{t}.$ With the mechanical-loss-related degradation including fatigue damage, lubrication failure of frictional damping increase, the rotor requires more torque to reach the rated speed. Therefore, we change parameter $B_{m}$ to simulate the mechanical degradation equivalently. In terms of electromechanical-conversion-related degradation, back EMF coefficient $K_{e}$ reflects the degrading flux linkage. Therefore, $K_{t}$ (equal to $K_{e}$) is selected as a system-level equivalent parameter to represent electromechanical-conversion-related degradation. To model the stochastic evolution of degradation-related parameters, we adopt a Wiener process based on Brownian motion [11] to parameter $B_{m}$ and $K_{t}$ with Eq. (10).

(10) $$\left\{ \begin{array}{c} B_{m}\left(t_{k}\right)=B_{m}\left(t_{k-1}\right)+2a\Delta t+\sigma_{1}Y\sqrt{\Delta t}\\ K_{t}\left(t_{k}\right)=K_{t}\left(t_{k-1}\right)+b\Delta t+\sigma_{2}Y\sqrt{\Delta t} \end{array}\right.$$

where the value of the coefficients is set as Table 7 and the initial value $B_{m0}∼N\left(0.0015,5\times 10^{-5}\right)$,$K_{t0}∼N\left(1.4,0.014\right)$ and Y∼N(0,1).

**Table 7:** The value of the parameter of the degradation for$B_{m}$ and $K_{t}$.

parameter	value
a	$6.531\times 10^{-8}$
b	$-1.714\times 10^{-3}$
$\sigma_{1}$	$2.5\times 10^{-6}$
$\sigma_{2}$	$2\times 10^{-4}$

To accurately approximate the degradation process of a real CMG, we develop a parameter identification method based on the Extended H∞ filter. First, by combining Eqs. (1), (2), and (5), we derive a simplified mechanistic model of the system:

(11) $$\left\{ \begin{array}{c} L\frac{di}{dt}=\frac{1}{2}U-ri-\frac{1}{2}K_{t}\omega \\ J\frac{d\omega }{dt}=K_{t}i-T_{L}-B_{m}\omega \end{array}\right.$$

This continuous-time model is then discretized using the forward Euler method, yielding the following time-discrete equations:

(12) $$\left\{ \begin{array}{c} i\left(k+1\right)=\frac{T_{S}}{2L}U\left(k\right)-\left(\frac{rT_{S}}{L}-1\right)i\left(k\right)-\frac{T_{S}}{2L}K_{t}\left(k\right)\omega \left(k\right)\\ \omega \left(k+1\right)=\frac{T_{S}}{J}K_{t}\left(k\right)i\left(k\right)-\frac{T_{S}}{J}T_{L}\left(k\right)-\left(\frac{B_{m}\left(k\right)T_{S}}{J}-1\right)\omega \left(k\right) \end{array}\right.$$

where $T_{S}$ is the sampling interval.

For the identification process, the input vector u(k), output vector y(k), and state vector x(k) are defined as:

(13) $$\left\{ \begin{array}{c} u\left(k\right)={\left[U\left(k\right)\;T_{L}\left(k\right)\right]}^{T}\\ y\left(k\right)={\left[i\left(k+1\right)\;\omega \left(k+1\right)\right]}^{T}\\ x\left(k\right)={\left[Li\left(k\right)\;J\omega \left(k\right)\;K_{t}\left(k\right)\;B_{m}\left(k\right)\right]}^{T} \end{array}\right.$$

**Fig. 15:**
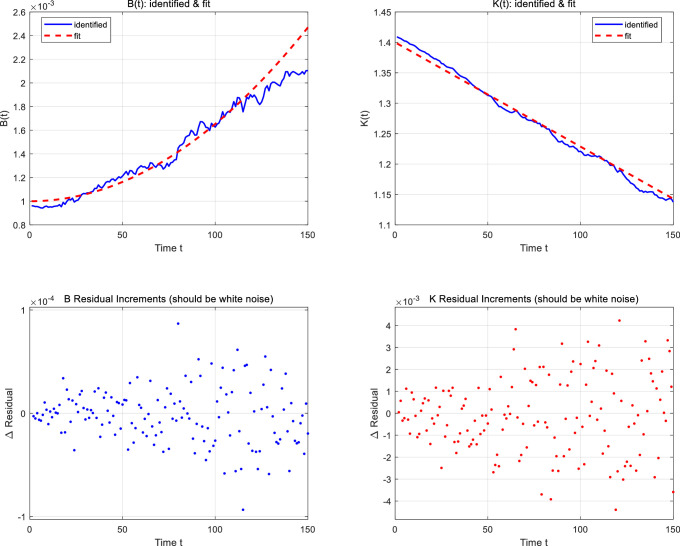
Identified $B_{m}$ and $K_{t}$ comparing with fit, and residual increments analysis.

**Fig. 16:**
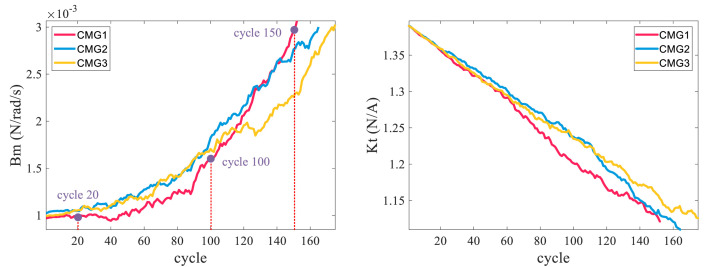
Degradation trend of the parameter $B_{m}$ and $K_{t}$.

The parameters $K_{t}$ and $B_{m}$ are identified using the Extended H∞ filter. To obtain a robust estimate, the final identified values for each cycle are taken as the average of the last 100 iterations of the filter. The parameters for the degradation process, presented in Table 7, are subsequently obtained by applying this identification procedure to each cycle of the real CMG flywheel system data. The process of parameter identification is shown in Fig 16, where the residual increments is nearly white noise. The results of Jarque-Bera normality test and Ljung-Box test are summarized in Table 8, where all p-values are larger than 0.05. Thus, the residual increments are normal without significant autocorrelation, consistent with the stochastic characteristics assumed by the Brownian-motion-based model. Fig. 15.

**Table 8:** The p-value of the parameter of the $B_{m}$ and $K_{t}$.

parameter	normality test p-value	Ljung-Box p-value
$B_{m}$	0.113675	0.277495
$K_{t}$	0.502137	0.068477

Fig 16 illustrates the degradation trends of the three CMG instances. These trends are consistent with those observed in other public datasets for RUL prediction, whose parameters decrease with a random noise. The end of life is declared when the parameter $B_{m}$ exceeds the empirical threshold of 0.003. This threshold should be interpreted as a dataset-specific, performance-oriented degradation criterion rather than a strict physical failure boundary. With the same threshold, since the rotational speed in OC2 is higher than that in OC1, the wear level is more severe, resulting in a shorter average lifespan (total number of cycles) for the CMG under this operating condition. In contrast, the load in OC3 is lower than that in OC2, leading to a slower degradation rate and, consequently, a significantly longer average lifespan for the CMG under OC3. These trends indicate that the generated lifetime data are consistent with the underlying physical mechanisms, thereby qualitatively validating the effectiveness of our data generation process.

**Fig. 17:**
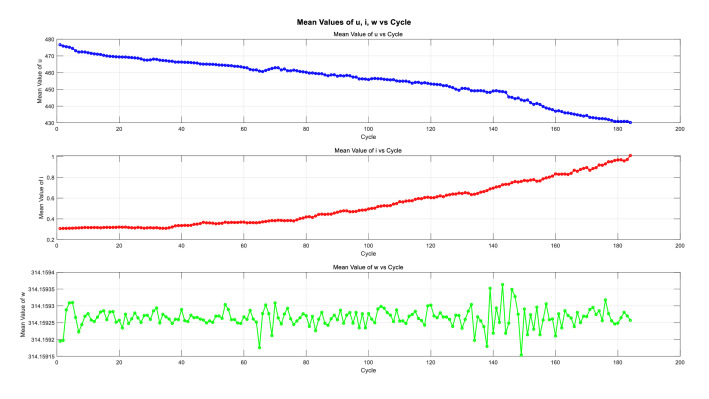
An example for degradation of voltage, current and rotational speed.

To illustrate the degradation trend in our dataset, samples from 013 in the OC1 set as an example are shown in Fig 17. The figure plots the mean values of current i, voltage u, and rotational speed w across all cycles. A clear trend can be easily observed in the mean current and voltage as degradation progresses. The decrease of $K_{t}$ implies that the motor generates less torque for the same current, thereby requiring a higher current to maintain the same load torque. Meanwhile, an increase in $B_{m}$ raises the frictional resistance, further elevating the torque demand. To compensate for the resulting torque deficit, the control system commands a higher current. However, because the motor operates under a closed-loop voltage-fed control scheme, the increased current leads to a larger voltage drop across the internal impedance (e.g., stator resistance and inductance), which in turn reduces the terminal voltage measured at the motor input. Consequently, we observe a simultaneous trend of rising current and falling voltage, both of which serve as indirect indicators of the underlying wear in $B_{m}$ and $K_{t}$. In contrast, the rotational speed remains relatively stable due to the robustness of the closed-loop control system.

### 4.11 Data generation

**Fig. 18:**
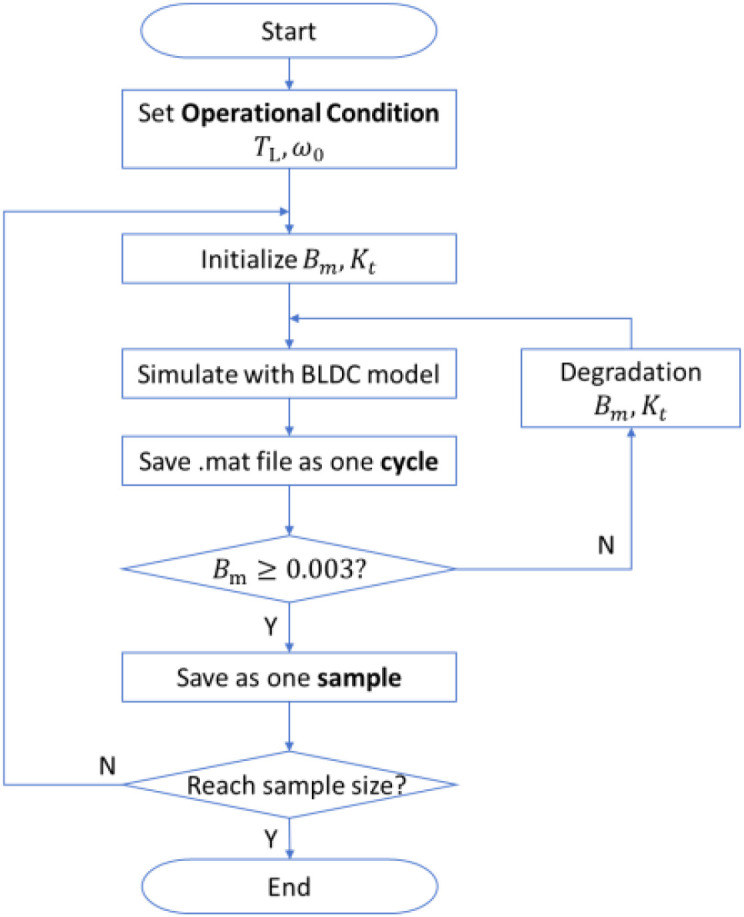
The workflow for generating the dataset.

The workflow for generating our dataset is illustrated in Fig 18. In the BLDC motor simulation, a nominal speed command is applied, and continuous small disturbances are added to the actual speed to emulate realistic speed fluctuations. Such fluctuations may arise from external operational disturbances, including spacecraft maneuvers and gimbal-related influences. The core objective of the flywheel system is to reject these disturbances and maintain the speed within a tight range around the given speed command value.

By changing the step input of the control system, the rotational speed command can be adjusted. And Load torque can be modified through changing the step input port *TL*.

The key configurations used in the Simulink simulation are summarized in Table 9.

**Table 9:** The key configurations used in the Simulink simulation.

Configuration Name	Configuration Value
Operation system	Windows 10
MATLAB version	2022a
Simulation step	5e-6s(fix-step)
Solver	ODE4
Random seed	rng('shuffle')

## Limitations

The first limitation is that the simultaneous motion of the gimbal system is not explicitly modeled. As a result, the present dataset is mainly suitable for degradation studies of the flywheel subsystem and may not fully capture certain failure signatures associated with gimbal-coupled dynamics.

The second limitation is that the control law includes several idealized assumptions and is not intended to exactly reproduce the controller implementation of an actual product. Nevertheless, under the operating conditions considered here, the flywheel generally operates near a constant speed with only small fluctuations, so this simplification is not expected to materially affect the degradation trends represented in the dataset.

The third limitation is that only two degradation-related parameters, $B_{m}$and $K_{t}$, are used to describe the degradation process. These two parameters are intended to capture the dominant mechanical-loss-related and electromechanical-conversion-related degradation behaviors considered in this work, but they do not exhaustively represent all possible degradation mechanisms.

The fourth limitation is that the end-of-life criterion $B_{m}>0.003$ was set empirically, because the available real CMG data do not include enough full run-to-failure observations.

A further limitation is that the full MATLAB/Simulink model is not currently released, and the use of rng('shuffle') does not guarantee strict run-to-run reproducibility, although it improves transparency of the simulation settings.

## Ethics Statement

For this research and analysis, no human or animal subjects are used and no data from social media platforms is used. The authors confirm that the provided dataset and presented work strictly meet the ethics requirements for publication in Data in Brief as mentioned in https://www.elsevier.com/de-de/researcher/author/policies-and-guidelines.

## CRediT Author Statement

**Diyin Tang:** Methodology, Writing; **Siyuan Liang:** Methodology, Writing, Experiment, Data curation; **Danyang Han:** Methodology, Experiment; **Bin Chen:** Visualization, Validation; **Jinsong Yu:** Supervision.

## Data Availability

Mendeley DataSimulation Degradation Datasets for Health Prognosis of a Control Moment Gyroscope's Flywheel system (Original data).
